# Statistical inference in brain graphs using threshold‐free network‐based statistics

**DOI:** 10.1002/hbm.24007

**Published:** 2018-02-15

**Authors:** Hugo C. Baggio, Alexandra Abos, Barbara Segura, Anna Campabadal, Anna Garcia‐Diaz, Carme Uribe, Yaroslau Compta, Maria Jose Marti, Francesc Valldeoriola, Carme Junque

**Affiliations:** ^1^ Medical Psychology Unit, Department of Medicine, Institute of Neuroscience University of Barcelona Barcelona Catalonia Spain; ^2^ Centro de Investigación Biomédica en Red sobre Enfermedades Neurodegenerativas (CIBERNED), Hospital Clínic de Barcelona Barcelona Catalonia Spain; ^3^ Movement Disorders Unit, Neurology Service, Hospital Clínic de Barcelona. Institute of Neuroscience, University of Barcelona Barcelona Catalonia Spain; ^4^ Institute of Biomedical Research August Pi i Sunyer (IDIBAPS) Barcelona Catalonia Spain

**Keywords:** connectomics, functional connectivity, network‐based statistic, structural connectivity, threshold‐free cluster enhancement

## Abstract

The description of brain networks as graphs where nodes represent different brain regions and edges represent a measure of connectivity between a pair of nodes is an increasingly used approach in neuroimaging research. The development of powerful methods for edge‐wise group‐level statistical inference in brain graphs while controlling for multiple‐testing associated false‐positive rates, however, remains a difficult task. In this study, we use simulated data to assess the properties of threshold‐free network‐based statistics (TFNBS). The TFNBS combines threshold‐free cluster enhancement, a method commonly used in voxel‐wise statistical inference, and network‐based statistic (NBS), which is frequently used for statistical analysis of brain graphs. Unlike the NBS, TFNBS generates edge‐wise significance values and does not require the a priori definition of a hard cluster‐defining threshold. Other test parameters, nonetheless, need to be set. We show that it is possible to find parameters that make TFNBS sensitive to strong and topologically clustered effects, while appropriately controlling false‐positive rates. Our results show that the TFNBS is an adequate technique for the statistical assessment of brain graphs.

## 1 INTRODUCTION

The brain is a complex network, and its function is dependent on the interactions between distributed regions (Sporns, 2016). In parallel with the development of novel acquisition and analytic strategies, interest in studying functional and structural brain networks through neuroimaging has greatly increased in the last few years in an attempt to understand the organizational principles underlying normal brain functioning (Mill, Ito, & Cole, 2017; Misic & Sporns, 2016), and the alterations underlying neural deficits in different pathological processes (Pievani, de Haan, Wu, Seeley, & Frisoni, 2011; Shi & Toga, 2017). One novel approach made possible by these recent technical improvements is the comprehensive description of brain functional or structural connections in the brain, that is, the connectome (Fornito, Zalesky, & Breakspear, 2015; Sporns, Tononi, & Kötter, 2005).

The study of large data sets such as the human connectome requires systematic approaches that provide relevant and reproducible parameters. In this context, graph theory, describing brain networks as a set of nodes interconnected by edges (characterized by structural or functional connections), has been widely adopted as a strategy for the study of these highly complex networks (Fornito, Zalesky, & Breakspear, 2013). Summary measures derived from graph theory provide information about whole‐brain or regional, node‐centric, topological network organization (Rubinov & Sporns, 2010). The connectome can also be analyzed at the connection level. Connection‐level group analyses have the desirable ability of describing local effects in the connectome, thus providing easily interpretable information that complements the more abstract graph‐theoretical topological parameters (Zalesky, Fornito, & Bullmore, 2010). This is especially interesting considering that certain brain regions are vulnerable to specific disorders (Meyer, 1936), implying that some portions of the connectome will be more vulnerable to specific pathological mechanisms than other regions. Topographical patterns of structural and functional connectivity disruption have indeed been shown to be associated with clinical phenotypes in diseases such as frontotemporal dementia and Alzheimer's disease (Pievani, Filippini, van den Heuvel, Cappa, & Frisoni, 2014). Connection‐wise approaches, however, are usually hindered by the high dimensionality of connectomics data sets. Conventional mass‐univariate testing repeated for every connection may suffer from low statistical power if appropriate correction for the high number of tests is performed (Fornito et al., 2013; Meskaldji et al., 2013).

Some neurobiological assumptions, however, may prove to be useful for the development of statistical methods for assessing brain graphs. Recent evidence indicates that connectivity disruptions in brain disorders progress along specific networks (Greicius & Kimmel, 2012; Jones et al., 2016). Even localized brain pathologies lead to more global network alterations (Stam, 2014), as might be expected in an interconnected system. Focal neuronal dysfunction can lead to changes in the firing patterns of postsynaptic cells, possibly manifesting as downstream functional connectivity alterations. Neuronal degeneration, on the other hand, can lead to deafferentation of its target cells and loss of trophic support to presynaptic neurons (Reier & Velardo, 2003), which might manifest as downstream and upstream structural connectivity abnormalities.

In the context of neurodegenerative diseases, furthermore, it is currently believed that the accumulation of toxic, misfolded proteins in highly susceptible neurons is followed by the transneuronal spread to other vulnerable cells to which they are connected (Campbell et al., 2015; Fornito et al., 2015; Raj, Kuceyeski, & Weiner, 2012; Saxena & Caroni, 2011). Analytical approaches with enhanced sensitivity to effects involving topologically neighboring connections might combine the ability to detect biologically plausible pathological alterations, while minimizing the possibility of finding disconnected, potentially spurious results related to the noisy nature of MRI measures used for connectivity estimation (Bright & Kevin, 2017; Liu, 2016; Polders et al., 2011; Zalesky & Fornito, 2009).

Different statistical methods have been developed to perform inference on brain graphs (Fornito et al., 2013; Meskaldji et al., 2013; Varoquaux & Craddock, 2013). One of the most frequently used edge‐wise methods is the network‐based statistic (NBS) (Zalesky et al., 2010). The NBS is designed to identify clustered effects in brain graphs, and has been used in different study settings (Abós et al., 2017; Cocchi et al., 2012; Conti et al., 2017; McColgan et al., 2017; Rigon, Voss, Turkstra, Mutlu, & Duff, 2017; Roberts et al., 2017a). Specifically, the NBS is a technique that aims to identify connected components, consisting of neighboring edges that display statistical effects above a predetermined threshold (Zalesky et al., 2010). The statistical significance of the connected components found is then established by estimating the likelihood of finding connected components with equal or greater extent (i.e., number of edges comprised in the connected component) or intensity (also considering test statistic values in the component) by chance. In the presence of statistical effects spanning adjacent edges, the NBS tends to show improved statistical power when compared with mass‐univariate testing controlling for multiple testing, while still providing control over the family‐wise error (FWE) rate in the weak sense (Meskaldji et al., 2013; Zalesky et al., 2010).

In this study, we describe a technique called threshold‐free network‐based statistics (TFNBS), designed to assess the presence of statistical effects in brain graphs. The TFNBS combines the NBS with a method commonly used in voxel‐wise analyses, threshold‐free cluster enhancement (TFCE) (Smith & Nichols, 2009). TFCE is a standard cluster‐defining approach employed by FSL (https://fsl.fmrib.ox.ac.uk/fsl/), one of the main software packages in neuroimaging. TFNBS enhances effects occurring in neighboring network edges, accounting for the topological dependency in the data, while maintaining sensitivity to strong localized effects and providing adequate control of the FWE rates. Our objective in this study is to test the TFNBS algorithm using different types of simulated data and different test parameters, with the goal of providing principled and evidence‐based criteria for parameter choice.

## 2 METHODS

To test the properties of the TFNBS algorithm, we use synthetic data containing different ground‐truth effects and data containing only random noise. As detailed below, the initial topological structure of the ground‐truth effects used was derived from the comparison between a group of healthy controls and a group of Parkinson's disease patients with mild cognitive impairment using resting‐state functional connectivity. In contrast with the original implementation of the NBS, TFNBS obviates the need for the a priori definition of a “hard” component‐defining threshold. Other parameters, however, need to be specified, as explained below.

### 2.1 TFNBS general algorithm

**Figure 1:**
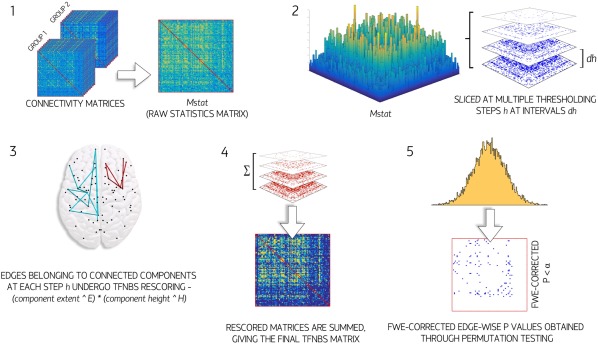
TFNBS algorithm. Initially, the raw *F* statistics matrix *Mstat* (Panel 1) is thresholded at a series of steps *h* (Panel 2). The step interval *dh* was defined as a hundredth of the maximum value in *Mstat*. At each thresholding step, suprathreshold connected components are identified (Panel 3). The value of each matrix element belonging to a connected component is replaced by the component's topological size (number of connections) raised to the power *E*, multiplied by the component's height (equal to the current threshold) raised to the power *H* (3). The matrices obtained at each step are subsequently summed, giving the final TFNBS score for every network edge (Panel 4). Statistical significance is established through permutation testing (Panel 5). At each permutation, group membership is shuffled across subjects, and the steps above are repeated. Raw statistics are obtained from the whole connectivity matrix at each permutation, thus preserving topological dependencies among connections. Whole‐connectome FWE‐corrected *p* values are obtained by comparing each connection's TFNBS score with the null distribution of maximal connectome‐wise scores at each permutation. Figure by Baggio, 2017; available at https://doi.org/10.6084/m9.figshare.5188753.v1 under a CC‐BY4.0 license [Color figure can be viewed at http://wileyonlinelibrary.com]

The first step of the TFNBS algorithm, as in NBS, is the computation of individual subjects' symmetric N×N connectivity matrices ***M***, where *N* is the total number of brain regions defined as nodes (Figure 1). Entry *i,j* in ***M*** describes the strength of structural or functional connection between nodes *i* and *j*, in a total of $N\times \left(N-1\right)/2$ unique internodal connection values if ***M*** is fully connected. Subsequently, the desired group test statistic is calculated for each entry, producing a group N×N raw test statistics matrix ***Mstat***.

In the NBS, a cluster‐defining threshold is then applied to ***Mstat***. Connections that survive this initial threshold are grouped with topologically neighboring suprathreshold edges into connected components. In the most commonly used variant of the NBS (NBS extent), the extent of each component (i.e., the number of connections comprised) is stored, and an FWE‐corrected *p* value is ascribed to each component through permutation testing (by comparing actual component sizes with the null distribution of sizes across permutations). Optionally, the magnitude of the effect of a component can be calculated using its intensity (sum of test statistic values across the edges comprised in the connected component—NBS intensity).

In the TFNBS, ***Mstat*** undergoes a transformation whereby the value of each edge is replaced by its TFNBS score (Figure 1). As this score is determined by the strength of statistical effect (height) at this connection and by the heights of its neighboring edges, final TFNBS scores will be influenced by how topologically “clustered” these effects are. Specifically, in the TFNBS transformation, several thresholds *h* are applied to ***Mstat***, from a baseline value up to the maximum height in the matrix. At each thresholding step, suprathreshold connected components are identified, and the values of all connections contained in a component are replaced by ${e(h)}^{E}\times h^{H}$, where *h* is the height of the current threshold, *e*(*h*) is the component size at threshold *h*, and *E* and *H* are the extension and height enhancement parameters, respectively. All other edges are set to zero. This results in an N × N × t matrix, where *t* is the number of thresholding steps. This transformed‐scores matrix is then summed across the third dimension, producing the N × N final TFNBS score matrix. The goal of this computation is to find the TFNBS‐transformed score of edge *i,j* by solving

$$TFNBS\left(i,j\right)=\int_{h=ho}^{h(i,j)}e{\left(h\right)}^{E}h^{H}dh$$

where *dh* is the interval between thresholding steps. In our algorithm, parameter *dh* was set at a hundredth of the maximum value in ***Mstat***. By setting *h0* to zero, therefore, ***Mstat*** undergoes 101 thresholding steps, whereas the number of steps in each ***Mstat‐rand*** varies according to its maximum height.

This nonlinear enhancement procedure is analogous to the implementation of TFCE for voxel‐wise analyses proposed by (Smith & Nichols, 2009). To adapt it to graph analyses, however, the definition of neighboring points at each thresholding step requires the identification of connected components, which in the NBS is only performed at the predefined threshold.

Through permutation testing, a *p* value can be ascribed to each entry in the TFNBS‐enhanced matrix. At each iteration of the permutation procedure, subjects can be randomly relabeled (e.g., by reshuffling group membership), and the resulting raw statistics matrix ***Mstat‐rand*** undergoes TFNBS scoring. The resulting *p* values can be corrected for multiple testing across the connectome (by comparing a connection's scores to the distribution of maximum scores across the matrix under the null hypothesis) or uncorrected (by comparing a connection's score to its own distribution of scores under the null).

### 2.2 Parameter selection

**Figure 2:**
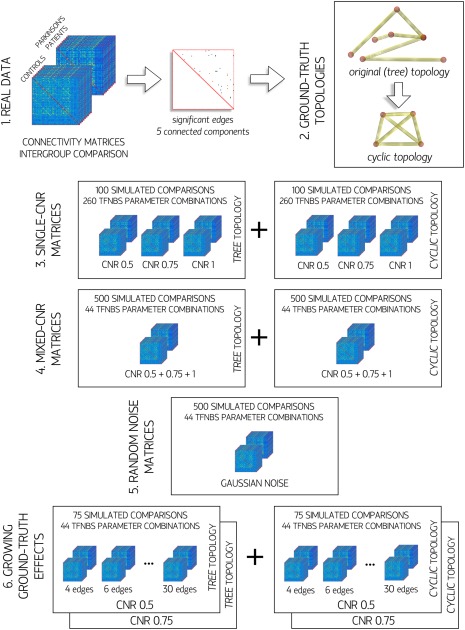
Schematic representation of analysis pipeline. Individual functional connectivity matrices of a sample of healthy subjects and patients with Parkinson's disease and mild cognitive impairment were initially generated. Intergroup comparisons (two‐tailed independent‐samples Student's *t* test, α = 0.003) were performed to define ground‐truth effect edges (Panel 1). Panel 2: Actual intergroup differences displayed a *tree* topology; additional ground‐truth connected components with identical number of edges but forming cliques were generated (*cyclic* topology). Panel 3: Simulated subject groups (consisting of 100 “normal” and 100 “reduced connectivity” subjects each) containing ground‐truth effects (*tree* or *cyclic* topology) with single contrast‐to‐noise ratios (CNR = 0.5, 0.75, or 1) were compared with TFNBS using 260 *E*/*H* parameter combinations. Panel 4: Simulated subject groups (100 “normal” vs 100 “reduced connectivity” subjects) containing ground‐truth effects (*tree* or *cyclic* topology) with mixed CNR (0.5, 0.75, and 1) were compared with TFNBS using a subset of 44 *E*/*H* parameter combinations. Panel 5: Simulated subject groups (200 individual matrices containing random noise) were compared using TFNBS (44 *E*/*H* parameter combinations). Panel 6: Simulated subject groups (100 “normal” and 100 “reduced connectivity” subjects) were generated, each with a single ground‐truth component with size ranging from 4 to 30 edges (at steps of 2), a single CNR (0.5 or 0.75), and either *tree (linear)* or *cyclic* topology. The number of simulated comparisons in each box refers to each of the conditions described therein [Color figure can be viewed at http://wileyonlinelibrary.com]

For three‐dimensional voxel‐wise analyses using TFCE, the recommended *E* and *H* parameters are 0.5 and 2, respectively (Smith & Nichols, 2009). Values that are appropriate for smoothed, three‐dimensional voxel matrices cannot be directly extrapolated to connectomics data, however. Here we initially test a range of 13 *E* (0.125 and 0.25–3 at intervals of 0.25) and 20 *H* parameter values (0.25–5 at intervals of 0.25), in a total of 260 *E*/*H* combinations. These combinations were tested using synthetic data with three different effect sizes and contrast‐to‐noise ratios (CNR), and two different types of topological organization, as described in the section *Initial parameter search* below. The results of the assessment of sensitivity and specificity of these initial simulations were then used to select a narrower range of *E* and *H* values, subsequently used to test simulated subject groups containing signal with different topologies and variable effect sizes/CNR (Section 3.2), and data containing only random noise to assess the occurrence of false positives in the absence of ground‐truth signals. These analyses were used to more accurately assess test properties such as sensitivity, specificity, number of false‐positives, and FWE rates. Figure 2 illustrates the analysis pipeline followed, described in detail below.

### 2.3 Ground‐truth effects matrix

We initially generated a ground‐truth matrix containing a pattern of connections defined as “altered,” to be used in the simulated connectivity matrices. To make these ground‐truth effects reflect a somewhat realistic topology, we performed univariate comparisons between a sample of Parkinson's disease patients with mild cognitive impairment (*n* = 27) and a group of healthy controls (*n* = 38), seen in a previous study by our group to have significant resting‐state functional connectivity differences (Abós et al., 2017). Data acquisition and image preprocessing were identical to those used in that study. FreeSurfer v5.1.0 (http://surfer.nmr.mgh.harvard.edu/) was used to divide the cerebral gray matter into 68 cortical and 14 subcortical regions based on the Desikan‐Killiany atlas (Desikan et al., 2006). Entry *i,j* in a subject's 82 × 82 connectivity matrix was defined by the correlation coefficient between the first eigenvariates of the time series of voxels contained in regions *i* and *j*.

**Figure 3:**
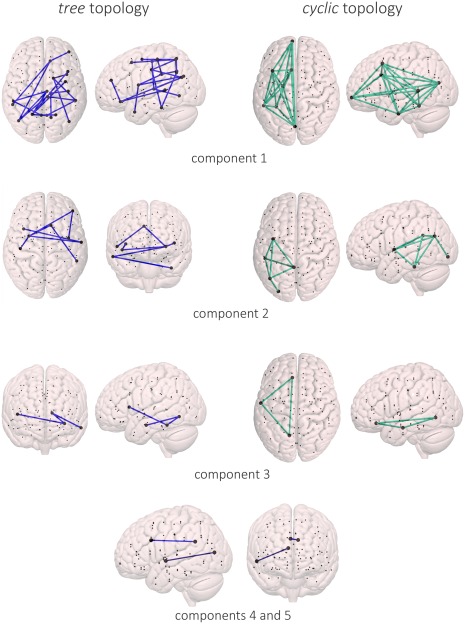
Ground‐truth connected components. Schematic anatomical representation of the five connected components used as ground‐truth effects in simulated data for the *tree* topology (left) and *cyclic* topology (right). Components *4* and *5*, each comprising a single edge, were identical in both topologies. Brain plots were created with Surf Ice (https://www.nitrc.org/projects/surfice/) [Color figure can be viewed at http://wileyonlinelibrary.com]

Two‐tailed independent‐samples *t* tests were then applied to each matrix entry, comparing the two subject groups. Connections that survived a liberal threshold of *p* < .003, uncorrected for multiple comparisons, were included in the ground‐truth matrix. This matrix finally included 32 “altered” connections, divided into five connected components: the largest containing 20 connections (linked to 20 nodes), the second with seven connections (linked to eight nodes), the third with three connections (linked to four nodes), and the two smallest with one connection each (Figure 3 shows the schematic representation of these components). These components were acyclic trees, that is, comprising linear and branching segments, but no cycles (i.e., the maximum number of paths of ground‐truth edges between any pair of nodes was one).

### 2.4 Simulated data

Three sets of synthetic data were generated:

1. Single‐CNR matrices: In this step, three sets of 100 simulated subject groups were created, with each group composed of 200 “subjects” divided into 100 “healthy subjects” and 100 “disrupted connectivity subjects.” Connectivity matrices were sized 82 × 82 and fully connected. Healthy subjects' matrices contained only noise (sampled from a normal distribution with mean $\bar{X}$ = 0 and standard deviation σ = 1). For subjects with disrupted connectivity, matrices contained Gaussian noise ( $\bar{X}$ = 0, σ = 1) in all connections except the 32 edges previously defined as ground‐truth effects. These ground‐truth‐signal connections were set by randomly selecting from a normal distribution of values with $\bar{X}$ = −0.5 (σ = 1) in the first set, $\bar{X}$ = −0.75 (σ = 1) in the second set, and $\bar{X}$ = −1 (σ = 1) in the third set. This procedure therefore produced effects with respective average CNR (as well as a Cohen's *d* effect size) of 0.5, 0.75, and 1.
  The TFNBS algorithm is sensitive to signal extent and intensity, not to the topological characteristics of the components detected at each step. As such, the final TFNBS score is not primarily determined by whether the effects display cyclic or tree topologies. The presence of noise, however, might have a differential impact on the detection of effects that do not contain cycles; the random reduction in statistical effect of a central edge in a linear segment can “break” a component into two smaller ones. If a similar effect reduction affects an edge contained in a cycle, on the other hand, the component will have its extent reduced by one edge, but will otherwise retain its extension.
  To assess the effect of signal organized in a topology containing cycles, we generated additional single‐CNR matrices, with identical effect sizes, number of components, and component extents, but with ground‐truth edges connected to the minimum number of nodes possible, forming cliques (see Figures 2−2 and 3; components 4 and 5, comprising a single edge each, were identical in the two cases). In the remainder of this article, these ground‐truth effects will be referred to as having a cyclic topology, as opposed to the tree topology derived from the comparison of actual healthy controls and Parkinson's disease patients.
2. Mixed‐CNR matrices: Here, 10^3^ data set groups containing true effects plus random noise were generated, each with 200 simulated 82×82 individual subject matrices. Again, 100 subjects corresponded to a “healthy” group, with data consisting of random noise (normally distributed, $\bar{X}$ = 0 and σ = 1). The remaining 100 subjects in each group consisted of disrupted connectivity subjects. The same ground‐truth effect structures defined above (32 edges distributed into five connected components—half groups with the original tree topology, and half with the cyclic topology) were used, but a different magnitude of connectivity reduction was assigned to each component. Edges in the largest component (Component 1) were randomly sampled from a normal distribution of values with $\bar{X}$ = −0.75 (σ = 1), yielding a mean CNR of 0.75; in Component 2, edge values were chosen from a normal distribution with $\bar{X}$ = −1 (σ = 1), with a mean CNR of 1; and edge values in Component 3 were sampled from a normal distribution with $\bar{X}$ = −0.5 (σ = 1), yielding a mean CNR of 0.5. Finally, the values for the two smallest components (each made up of a single edge) were randomly sampled from normal distributions, Component 4 with $\bar{X}$ = −1 (σ = 1), and Component 5 with $\bar{X}$ = −0.5 (σ = 1), with respective CNR of 1 and 0.5.
3. Random‐noise matrices: In this step, 500 subject groups were generated, each with 200 subjects. Individual data consisted of 82×82 matrices with values randomly sampled from a normal distribution with $\bar{X}$ = 0 and σ = 1.
4. Growing ground‐truth component matrices: Finally, we systematically assessed the properties of the NBS using simulated data sets, consisting of 82×82 matrices containing random noise, and ground‐truth components with sizes ranging from 4 to 30 edges (at steps of 2). These ground‐truth components displayed CNR of either 0.5 or 0.75, and linear (tree) or cyclic (forming cliques) topologies. For each CNR, ground‐truth component size, and ground‐truth topology, 75 simulated group matrices were generated, in a total of 4,200 comparisons.

### 2.5 Statistical testing

For statistical inference, permutation testing (3,000 permutations) was performed using the *F* statistic obtained through a general linear model (GLM). At each permutation, subjects' group membership was randomly reshuffled and the *F* statistic was computed by comparing the 100 subjects assigned to the “healthy” group and the 100 subjects assigned to the “disrupted connectivity” group. In the analysis of random‐only data, half of the 200 subjects were randomly assigned to each group. Edges that displayed FWE‐corrected *p* values below the significance threshold of .05 were considered as positive results.

To assess the test properties, the N×N matrices ***P*** containing significant results (where edges surviving the statistical significance threshold were coded as 1, and all other edges as 0) and the ground‐truth matrices ***T*** (where ground‐truth edges were coded as 1 and all other edges as 0) were used to calculate the following parameters for each simulated group comparison:

- $Number\;of\;false\;positives\;\left(FP\right)=\;\sum_{i=1}^{N}\sum_{j=i}^{N}\left(\left(1-\;T_{ij}\right)\times P_{ij}\right)$
- $Number\;of\;false\;negatives\;\left(FN\right)=\;\sum_{i=1}^{N}\sum_{j=i}^{N}\left(T_{ij}\times \left(1-P_{ij}\right)\right)$
- $Number\;of\;true\;positives\;\left(TP\right)=\;\sum_{i=1}^{N}\sum_{j=i}^{N}\left(T_{ij}\times P_{ij}\right)$
- $Number\;of\;true\;negatives\;\left(TN\right)=\;\sum_{i=1}^{N}\sum_{j=i}^{N}\left(\left({1-T}_{ij}\right)\times \left(1-\;P_{ij}\right)\right)$
- $Sensitivity=\;\frac{TP}{TP+FN}$
- $Specificity=\;\frac{TN}{TN+FP}$
- False positive rate= 1−specificity
- FWE rate: a family‐wise error was defined as the presence of one or more false‐positive edges across the connectivity matrix. For each simulated group comparison, therefore, FWE = 0 or FWE = 1.

Average sensitivity, specificity, false‐positive rates, and FWE rates were calculated by averaging across corresponding simulated group comparisons (e.g., across the 500 mixed‐CNR group comparisons with the tree topology). Results are shown as curves plotting sensitivity against false‐positive rates and sensitivity against FWE rate, parameterized by *E*/*H* combination for TFNBS and primary *F*‐threshold for NBS. We choose this presentation over traditional receiver operating characteristic curves to facilitate the comparison of test properties across the range of parameters that need to be set a priori.

## 3 RESULTS

### 3.1 Initial parameter search

**Figure 4:**
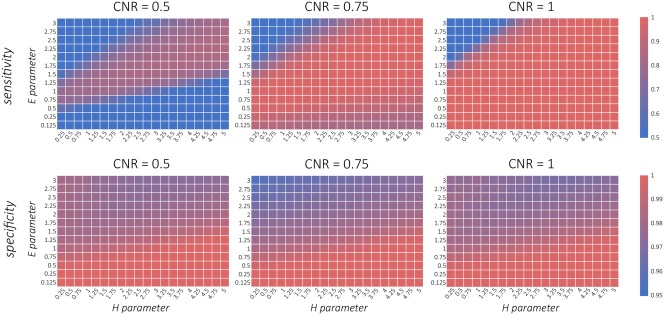
Initial parameter search. Heatmaps display mean sensitivity and specificity levels for each of the 260 *E*/*H* parameter combinations for the simulated data with the *tree* topology, with contrast‐to‐noise ratios (CNR) of 0.5 (left panels), 0.75 (middle panels), and 1 (right panels) [Color figure can be viewed at http://wileyonlinelibrary.com]

This initial analysis was performed using the single‐CNR matrices, and was designed to assess the impact of different *E* and *H* parameter combinations on sensitivity and specificity to statistical effects with different magnitudes. As expected, the presence of stronger effects was associated with higher sensitivity. Specificity also tended to be higher in data sets with larger effect sizes. Figure 4 shows the sensitivity and specificity of different *E*/*H* combinations in the three CNR levels tested using the tree ground‐truth topology. Increasing *H* and decreasing *E* led to more conservative hypothesis testing, that is, higher specificity and lower sensitivity. A similar overall pattern was observed with the cyclic topology (Supporting Information, Figure 1). For subsequent analyses, we aimed to select a range of *E*/*H* parameter combinations that were less vulnerable to false‐positives. We therefore chose a set of 44 combinations of conservative parameters, consisting of lower *E* and higher *H* values (*E* parameter range: 0.25–1, at intervals of 0.25; *H* parameter range: 2.25–4.75, at intervals of 0.25), to be further assessed in the next section.

**Figure 5:**
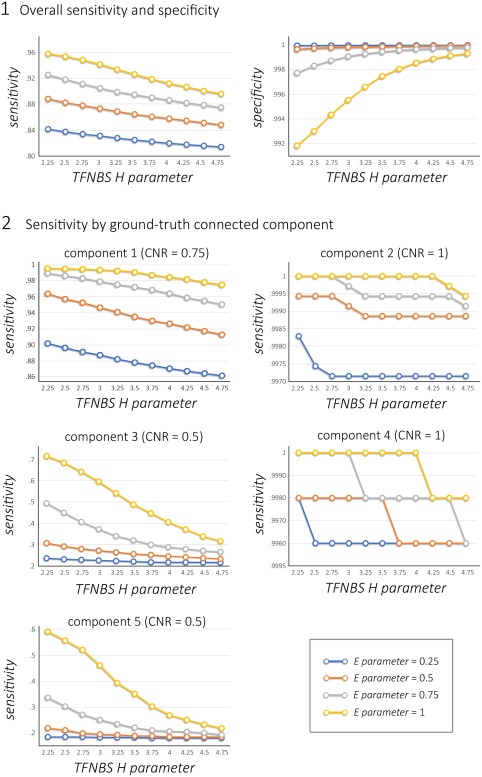
Mixed‐CNR matrices: TFNBS sensitivity and specificity analysis *(*tree *topology)*. Panel 1: Curve plots show mean sensitivity (left) and specificity (right) for the four TFNBS *E* parameter values assessed as a function of *H* value, across all ground‐truth edges. Panel 2: Plots show the sensitivity to edges in each of the five ground‐truth connected components, for the four *E* parameter values assessed as a function of *H* value. Components' contrast‐to‐noise ratios (CNR) are indicated. The *y*‐axes have been rescaled according to the range of values displayed [Color figure can be viewed at http://wileyonlinelibrary.com]

**Figure 6:**
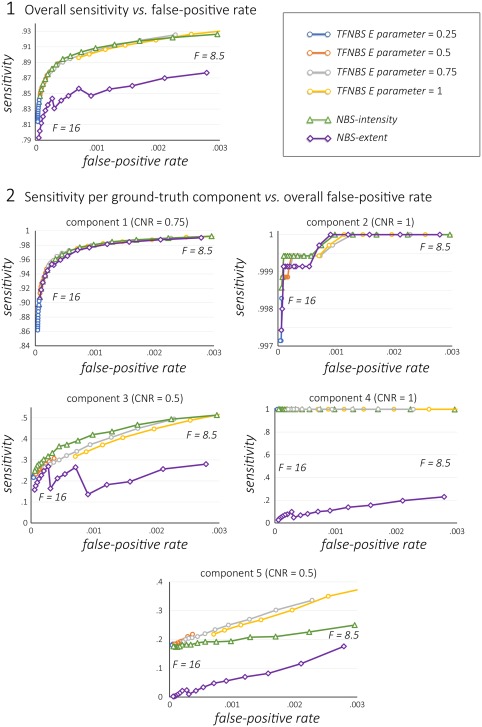
Mixed‐CNR matrices. TFNBS and NBS: sensitivity and specificity analysis (tree *topology)*. Panel 1: Curve plot shows mean sensitivity as a function of the mean false‐positive rate (*1‐specificity*) across all ground‐truth edges. Panel 2: Curves show mean sensitivities for each of the five ground‐truth connected components as a function of the mean overall false‐positive rate, for the four *E* parameter values assessed across 11 different *H* values. Components' contrast‐to‐noise ratios (CNR) are indicated. Curves marked with circles are parameterized by TFNBS *E*/*H* parameter value combinations. Each curve represents a different *E* parameter, and each point along the curves indicates a different value of *H*. Some parameters (*E* = 1 combined with *H* < 3.5) yielded specificities <0.997 and are not shown. Curves marked with triangles or diamonds indicate values obtained with the two tested variants of the NBS, parameterized by the *F*‐thresholds that displayed specificities ≥0.997. The *y*‐axes have been rescaled according to the range of values displayed [Color figure can be viewed at http://wileyonlinelibrary.com]

**Figure 7:**
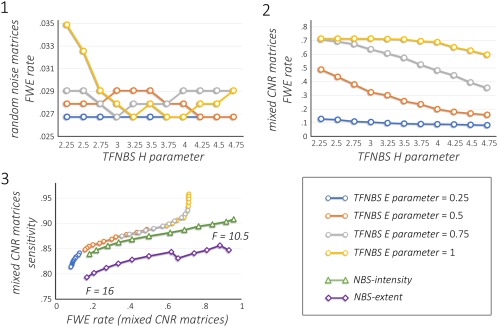
TFNBS and NBS family‐wise error rates. Panels 1 and 2 show mean family‐wise error (FWE) rates obtained using TFNBS with *random‐noise matrices* (Panel 1) and with *mixed‐CNR* tree *topology matrices* (Panel 2). Different curves depict different *E* parameter values, as a function of *H* parameter values. Panel 3: Relationship between sensitivity and FWE rates, obtained with mixed‐CNR matrices and *tree* topology. Curves marked with circles are parameterized by TFNBS *E*/*H* parameter combinations, and curves marked with triangles or diamonds are parameterized by the 12 NBS *F*‐thresholds (10.5–16) that yielded similar FWE rates [Color figure can be viewed at http://wileyonlinelibrary.com]

### 3.2 Focused parameter assessment

In this step, the mixed‐CNR matrices and random‐noise matrices were analyzed with TFNBS using the 44 *E*/*H* parameter combinations described above. The same data were also assessed with NBS, using 25 component‐defining *F*‐statistic thresholds (4–16, at intervals of 0.5, corresponding to *p* values of 0.0469–0.0001, respectively). Figure 5‐1 shows the overall specificity and sensitivity, averaged across all edges in the five ground‐truth connected components with a tree topology, for the four *E* parameters tested as a function of *H*. Again, lower *E* values and higher *H* values led to stricter testing. Supporting Information, Figure 2 shows the corresponding results for the cyclic ground‐truth pattern. With this topology, overall differences between more liberal and more conservative *E*/*H* combinations tended to be smaller than with the cyclic topology. This was mainly due to lower sensitivities and higher specificities at higher *E* combined with lower *H* combinations.

Sensitivity per connected component was, as expected, strongly determined by the component's CNR (Figure 5‐2 and Supporting Information, Figure 2‐2). Sensitivity to the two components with the strongest effects (Components 2 and 4, CNR = 1; and Component 1, CNR = 0.75) was high. Sensitivity to Components 3 and 5, both with CNR = 0.5, on the other hand, was considerably lower. Again, these weaker effects were more consistently detected when clustered in a larger component (Component 3) than when organized as a single connection (Component 5).

Figure 6‐1 (tree topology) and Supporting Information, Figure 3 (cyclic topology) show overall sensitivity as a function of the false‐positive rate for the different *E*/*H* combinations tested, and for the NBS across the component‐defining *F* thresholds that displayed similar specificities. For parameters with similar specificities across techniques, the TFNBS reached higher sensitivities than NBS‐extent; NBS‐intensity, on the other hand, displayed an overall sensitivity similar to that seen with the TFNBS for parameters with comparable specificities (Figure 6‐1 and Supporting Information, Figure 3‐1).

Figure 6‐2 and Supporting Information, Figure 3‐2 illustrate the relationship between component‐wise sensitivity and false‐positive rates obtained with NBS and TFNBS. For parameters with specificities higher than 0.997, sensitivity tended to be higher with the TFNBS and NBS‐intensity (*F*‐thresholds between 8.5 and 16). As expected, considering how NBS‐extent is designed, sensitivity (with *F*‐thresholds 8.5–16) was very low (<0.1) to the two components comprised of a single edge (Components 4 and 5), using this technique; the TFNBS and NBS‐intensity, on the other hand, showed high average sensitivities (between 0.996 and 1; and between 0.990 and 0.994, respectively) to Component 4 (CNR = 1) in all *E*/*H* combinations, whereas for Component 5 (CNR = 0.5) sensitivity ranged between 0.18 and 0.59 for TFNBS, 0.18 and 0.25 for NBS‐intensity, and 0.002 and 0.18 for NBS‐extent.

For the analysis of FWE rates, three sets of simulated data (random‐noise matrices, and mixed‐CNR matrices with tree and cyclic topologies) were analyzed. To define the FWE rate across the connectome, the presence or absence of any false‐positive connections at each comparison was registered. Averages across the 500 comparisons for each *E*/*H* parameter combination are shown in Figure 7−1 and −2. Average FWE rates in the data containing only random noise were inferior to 0.035 at the 44 *E*/*H* combinations assessed. FWE rates for the NBS, with F‐threshold between 8.5 and 16, were 0.0375–0.0425 (NBS‐intensity) and 0.0373–0.0020 (NBS‐extent).

FWE rates found when analyzing the mixed‐CNR matrices were notably higher, surpassing the 0.05 threshold at most of the parameters assessed with the TFNBS, and all parameters assessed with the NBS (Figure 7−2 and −3 and Supporting Information, Figure 4). With the TFNBS, these FWE rates were dependent on and positively related to the *E* parameter, and negatively related to the *H* parameter.

The simulated matrices used in this study are fully connected and noisy—as functional connectivity matrices often are. In this scenario, edge *i,j* in a true‐effect connected component with height *hc*, at a given slicing threshold *h* ≤ *hc*, can potentially be connected to up to N – (nc + 1) other nodes by chance (*nc* being the number of nodes to which node *i,j* is connected in the true‐effect connected component, and *N* is the total number of network nodes). If one considers a connected component such as tree‐topology Component 2, with 8 nodes and 7 intervening links (Figure 3), the total number of potential neighboring false‐positives in an 82‐node matrix is thus 634. Given that the probability of any such noise‐only connections, sampled from a normal distribution, to display an effect size ≥ 0.5 in a simulated subject group is $∼3.33\times 10^{-4}$, the probability of such a “spurious” effect to be a neighbor of a node in Component 2 is ∼0.21. This probability grows linearly with the number of nodes in the component (provided that the component contains no cycles) and reaches ∼0.53 for a component as large as Component 1. When the number of nodes in the component is reduced, even if the number of ground‐truth edges remains the same (as in the cyclic topology), the FWE rate becomes smaller (Figure 7−2 and −3 and Supporting Information, Figure 4).

Due to factors inherent to the type of data, the risk of family‐wise errors is therefore high when true effects are present in noisy connectivity matrices. The probabilities given above for a fully‐connected matrix, however, describe the “worst case scenario.” When matrices are originally sparse or are thresholded prior to statistical testing, as in structural connectivity studies (Roberts, Perry, Roberts, Mitchell, & Breakspear, 2017b; Zalesky et al., 2016) and sometimes in functional connectivity assessments (Yang et al., 2017), the reduced network density would decrease the risk of false‐positives. Even considering the probabilities described above, however, whenever any false‐positives occur, the total number of false‐positives should be small. As shown in Supporting Information, Figure 5, this was usually the case; except when *E* parameter = 1, the average number of false‐positives (in the comparisons that displayed at least one false‐positive using the mixed‐CNR matrices) tended to be below six for the tree topology and below two for the cyclic topology. For parameters with comparable sensitivities, the NBS (especially NBS‐extent) tended to yield higher FWE rates (Figure 7‐3 and Supporting Information, Figure 4‐2) and more false‐positive edges (Supporting Information, Figure 5‐3 and −4) than the TFNBS in matrices containing true statistical effects.

**Figure 8:**
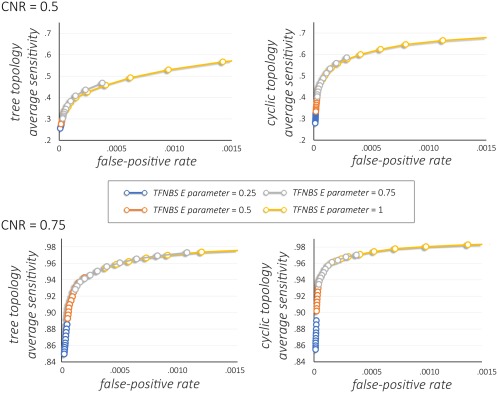
Growing ground‐truth components: sensitivity and specificity analysis. Random‐noise matrices containing ground‐truth connected components of sizes ranging from 4 to 30 edges (at steps of two) were assessed, with two different effect sizes (CNR = 0.5, top row; and CNR = 0.75, bottom row) and two topologies (linear *tree* topology, left, and cyclic topology, right). Plots represent the relationship between sensitivity and false‐positive rates (*1‐specificity*) averaged across networks with ground‐truth connected components of different sizes, parameterized by TFNBS *E*/*H* parameter combinations. Combinations consisting of *E* parameter value = 1 and low values of *H* yielded specificities below 0.9985 and are not shown [Color figure can be viewed at http://wileyonlinelibrary.com]

Finally, we systematically assessed the properties of the TFNBS with ground‐truth effects of variable extension, topology (cyclic and tree), and effect sizes (CNR of 0.5 and 0.75) using the growing ground‐truth component matrices. Figure 8 shows the relationship between true‐positive and false‐positive rates averaged across the 14 evaluated component extensions (4–30 edges, at steps of 2), using 44 *E*/*H* parameter combinations. Supporting Information, Figures 7 and 8 show the results for each component size. As expected, sensitivity to effects organized in a cyclic topology was higher than to linear effects; this difference was more evident in the matrices with the weaker effect size. Also, it again becomes clear that *E* parameter values of 0.25 and 1 were, respectively, highly conservative and highly liberal.

## 4 DISCUSSION

In this study, we assess the test properties of the TFNBS algorithm using different parameters and in different types of simulated data. TFNBS is a technique that enhances topologically clustered effects without requiring a component‐defining threshold. Our results show that TFNBS is sensitive to statistical effects clustered into connected components and to strong isolated signals, and appears to be a suitable statistical approach for statistical inference in brain connectivity graphs.

The widely used NBS is a technique designed to be sensitive to statistical effects that are organized into connected components in connectivity matrices (Zalesky et al., 2010). This aspect renders NBS more powerful than edge‐wise mass‐univariate analyses followed by correction for multiple testing (such as control of the false‐discovery rate; Benjamini, 2010), provided that effects are clustered (Zalesky et al., 2010). As shown in this study, the test properties of NBS are strongly dependent on the a priori component‐defining threshold, which is often chosen arbitrarily. Also, because inference is performed at the component level, edge‐wise significance levels are not obtained. NBS is therefore analogous to cluster‐based thresholding as used in voxel‐based neuroimaging analyses. The TFNBS, on the other hand, is analogous to TFCE. Similar to TFCE (Smith & Nichols, 2009), the TFNBS has the advantage of producing point‐wise *p* values, and preserving local maxima—retaining finer topological information than the NBS—without the need to set a component‐defining threshold.

Nonetheless, the adequacy of the TFNBS for assessing brain graphs cannot be directly extrapolated from the demonstrated usefulness of TFCE in voxel‐wise analyses. Three‐dimensional voxel‐wise data are fundamentally different from connectivity matrices—especially, for the topic at hand, concerning the definition of a point's neighbors. A cuboid voxel's potential neighborhood is defined by physical adjacency, with upper limits usually ranging from 6 (if only adjacent faces are considered) to 26 (if neighborhood can be established by adjacent faces, edges, or vertices) voxels. By contrast, node *i* in a connectivity matrix can potentially be connected to any other network node. As TFNBS scores are determined not only by an edge's height (i.e., magnitude of the statistic), but also by component extension (i.e., the heights of its neighboring edges), without discarding subthreshold effects, signal bleeding from true‐effect edges may affect extended areas of the network.

In the last few years, the issue of replicability and reproducibility in neuroimaging studies has gained considerable and justified interest (Poldrack et al., 2017). In this context, the adoption of statistical methods that maximize both sensitivity and specificity is critical (Bennett, Wolford, & Miller, 2009). In this study, we demonstrate that the test properties of the TFNBS are strongly influenced by the choice of parameters for the enhancement of signal height (*H* parameter) and component extent (*E* parameter), and that it is possible to find parameter combinations that display high specificity while maintaining sensitivity to effects that are topologically clustered, or that have large magnitudes.

The initial parameter search, exploring a wide range of *E* and *H* parameters in different CNR and topology settings, showed that the combination of lower *E* and medium to large *H* values led to conservative testing, but with high global sensitivity to effects with high CNR. Further assessment of a more restricted range of *E*/*H* combinations (*E* values between 0.25 and 1, and *H* values between 2.25 and 4.75) showed that higher *E* values yield higher power, at the cost of more false‐positive edges. With the simulated data used in the mixed‐CNR matrices, high specificities could be obtained with all but the highest *E* parameter value tested.

The assessment of sensitivity as a function of component extent and height, using data containing signals with different CNR, confirmed that TFNBS power is determined by both aspects (extent and magnitude) of the ground‐truth effects. Sensitivity to low‐CNR effects (CNR = 0.5) tended to be below 0.5 for the component sizes tested (with the exception of the excessively liberal *E* = 1). At higher CNR (0.75 and 1), detection power was very high while still maintaining low false‐positive rates. Comparison with the NBS shows that NBS‐intensity produces an overall similar sensitivity versus false‐positive rate curve to that obtained with the TFNBS. NBS‐extent, as expected, displayed very low sensitivity to effects organized into small connected components, even at high CNR.

The greater sensitivity of the TFNBS (and NBS‐intensity) to detect isolated edges is desirable if one assumes that effects with this topology are neurobiologically plausible, an assumption that is difficult to test formally. In the case of “small” networks, where nodes are defined, for example, by a few intrinsic connectivity networks, this ability is likely to be of interest. Irrespective of this, a significant single‐edge finding in the TFNBS can in fact be the reflection of a more extended underlying signal. That is, it is possible for a single connection to be identified as statistically significant due to the contribution of a broader supporting component that nonetheless exceeds a given alpha level at a single point of the matrix (as “the tip of the iceberg”). A second issue is a caveat to the sensitivity of the TFNBS to isolated effects. Brain connectivity graphs—either functional or structural—are inherently noisy. Low signal‐to‐noise ratios may lead to strong group effects arising in a few isolated connections by chance. If TFNBS is sensitive to such spurious effects, it might be vulnerable to false‐positive findings. Analysis of matrices containing only random noise, however, showed that the TFNBS offers adequate control of the FWE rate when no true effects are present.

Analyzing data containing mixed‐CNR ground‐truth effects plus random noise, for the range of high‐specificity test parameters assessed, TFNBS and NBS FWE rates were similar. Nonetheless, the TFNBS showed higher specificity and lower FWE rates than the NBS for parameters with comparable sensitivity. This was especially true when compared with NBS‐extent, but also with NBS‐intensity in the case of the tree topology. These findings indicate that, as expected, the presence of a noisy background does lead to false‐positives; nonetheless, for parameters with similar sensitivities, the “signal bleeding” potentially caused by the TFNBS procedure does not increase the FWE rate or reduce the test specificity compared with the NBS. Regarding the effects of topology on TFNBS and NBS test properties, the higher specificities and lower FWE rates observed with the cyclic topology emphasize the relationship between the number of nodes linked to “actual” effects and the occurrence of false positives, as described above. The spatial specificity of the TFNBS can therefore be reduced if effects involve a high proportion of network nodes, especially if fully connected matrices are used. Nonetheless, the edge‐wise *p* values produced with the TFNBS allow exploring the data a posteriori as information regarding the relative significance of effects is retained in the statistical significance matrices.

One limitation of this study is the fact that edge values in the simulated connectivity matrices were selected randomly from a normal distribution. These networks therefore do not replicate the topological properties of actual human functional connectivity matrices such as transitivity, modular structure, or small‐world and scale‐free characteristics (Stam, 2014; Zalesky, Fornito, & Bullmore, 2012). Considering that we assessed the effect of randomly distributed noise added to the connectivity matrices, the underlying topology is unlikely to have an impact on the results of the simulated data set analyses. Simulated data incorporating organized noise, nonetheless, might provide useful information regarding its effects on the sensitivity to ground‐truth effects. Additionally, the inclusion of other methods for statistical inference on brain graphs, although outside the scope of this study, might have provided a more thorough comparative assessment of currently available techniques.

In summary, we have demonstrated that the TFNBS algorithm can be a valid approach for performing statistical inference on brain graphs. Comparisons with the method it is based on, the NBS, reveal that the TFNBS may display statistical power similar to the NBS‐intensity variant, with possible advantages regarding FWE rates and number of false‐positive connections. The main advantage, however, is the possibility of assigning edge‐wise *p* values. Results obtained with different TFNBS parameters show that high test specificity can be obtained using *E* parameter values <1 alongside *H* parameter values ≥2.25. Statistical power, nonetheless, may be too low with values of *E* < 0.5. We therefore recommend *E* parameter values of 0.5 (combined with *H* parameter values between 2.25 and 3) or 0.75 (combined with *H* parameters between 3 and 3.5).

AA was supported by an FI‐DGR grant. BS, HCB, AIGD, CJ, YC, MJM, and FV report no disclosures.

## Supporting information

Additional Supporting Information may be found online in the supporting information tab for this article.

Supporting Information
