# Supplementary material for: Statistical inference in brain graphs using threshold‐free network‐based statistics
Source: Hum Brain Mapp. 2018 Feb 15;39(6):2289–302. doi: 10.1002/hbm.24007 (PMC6619254; doi:10.1002/hbm.24007)
Supplement: Supplementary file 1 — Supporting Information [file HBM-39-2289-s001.pdf]

Supporting Information

Statistical inference in brain graphs using threshold-free network-based statistics

Hugo C. Baggio[1]*, Alexandra Abos[1]*, Barbara Segura[1], Anna Campabadal[1], Anna Garcia-Diaz[1], Carme Uribe[1], Yaroslau Compta[2,3], Maria Jose Marti[2,3], Francesc Valldeoriola[2,3], Carme Junque[1,2,4]

[1]Medical Psychology Unit, Department of Medicine. Institute of Neuroscience, University of Barcelona. Barcelona, Catalonia, Spain.

[2]Centro de Investigación Biomédica en Red sobre Enfermedades Neurodegenerativas (CIBERNED), Hospital Clínic de Barcelona. Barcelona, Catalonia, Spain.

[3]Movement Disorders Unit, Neurology Service, Hospital Clínic de Barcelona. Institute of Neuroscience, University of Barcelona, Barcelona, Catalonia, Spain.

[4]Institute of Biomedical Research August Pi i Sunyer (IDIBAPS). Barcelona, Catalonia, Spain.

*AA and HCB contributed equally to the manuscript.

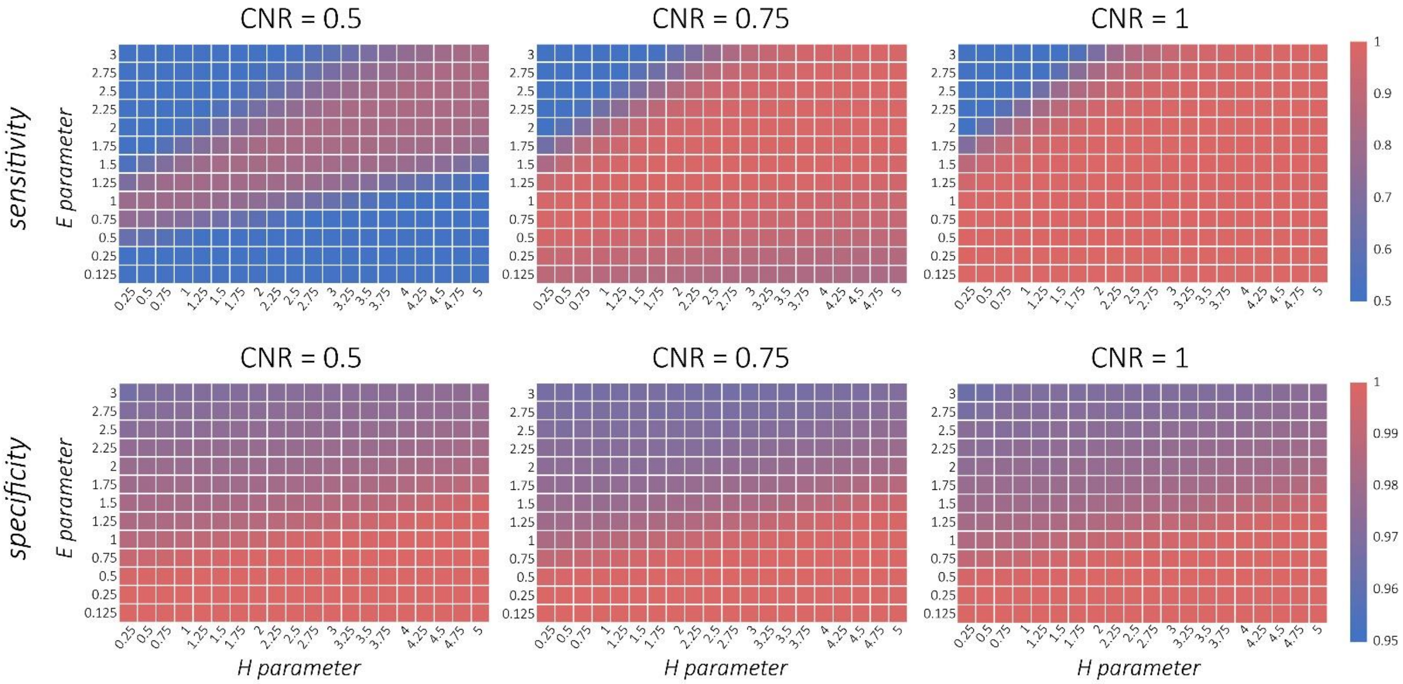

Supporting Information, Figure 1. Initial parameter search – ***cyclic topology***. Heatmaps display mean sensitivity and specificity levels for each of the 260 *E*/*H* parameter combinations for the simulated data with the *tree* topology, with contrast-to-noise ratios (CNR) of 0.5 (left panels), 0.75 (middle panels), and 1 (right panels).

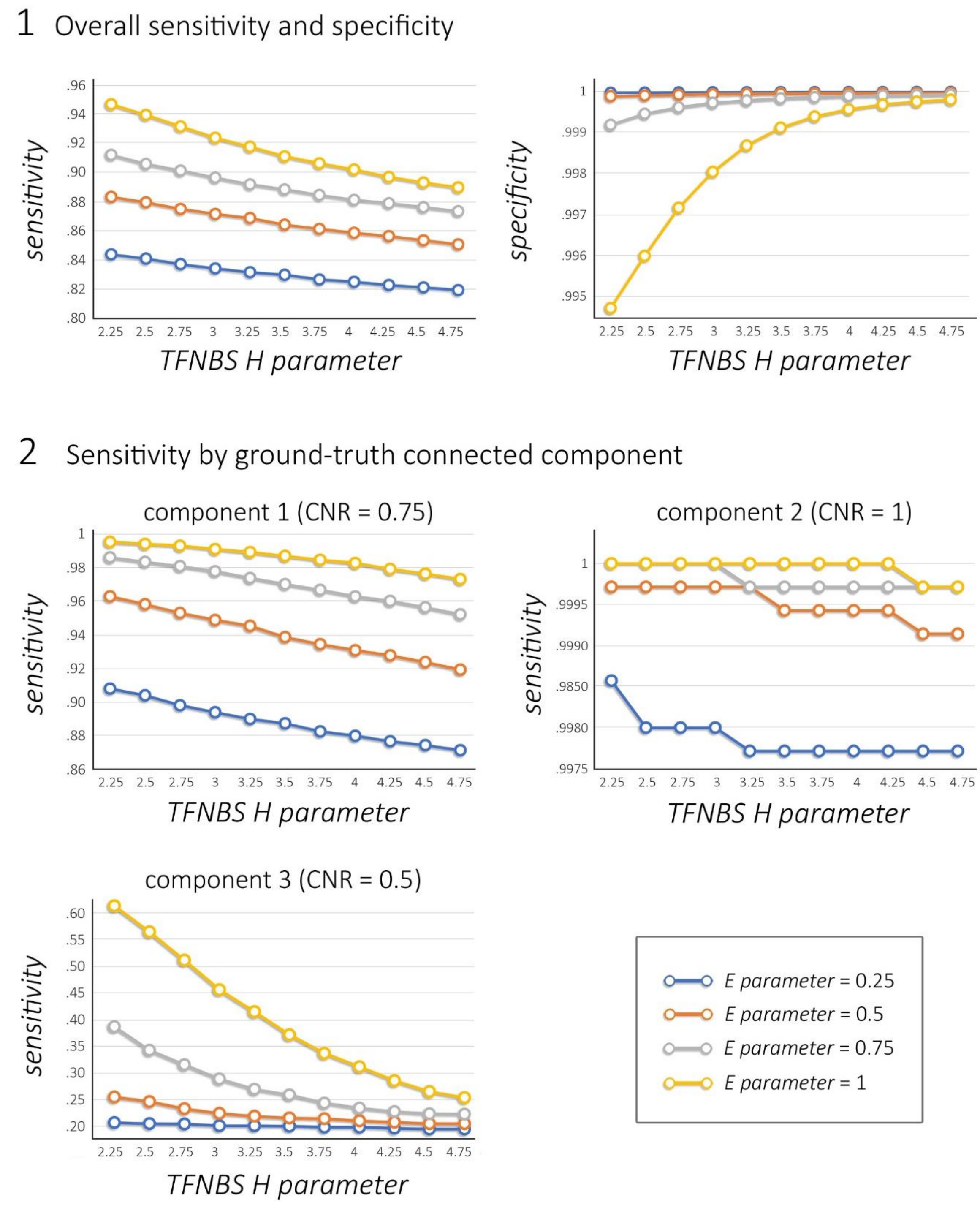

*Supporting Information, Figure 2. Mixed-CNR matrices – TFNBS sensitivity and specificity analysis (***cyclic topology***).*
Panel 1: Curve plots show mean sensitivity (left) and specificity (right) for the four TFNBS *E* parameter values assessed as a function of *H* value, across all ground-truth edges. **Panel 2:** Plots show the sensitivity to edges in each of the five ground-truth connected components, for the four *E* parameter values assessed as a function of *H* value. Components' contrast-to-noise ratios (CNR) are indicated. The *y* axes have been rescaled according to the range of values displayed.

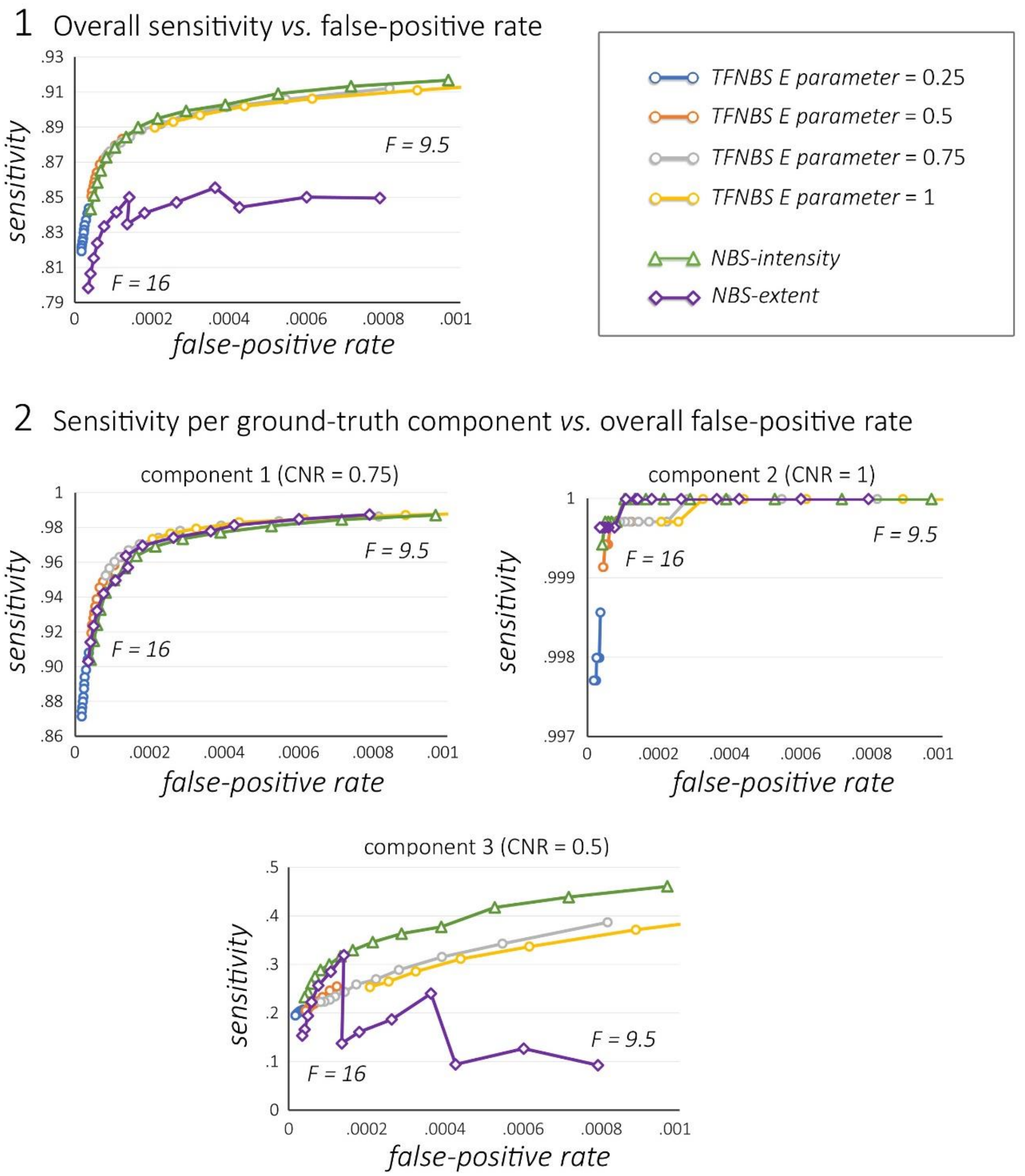

*Supporting Information, Figure 3. Mixed-CNR matrices. TFNBS and NBS – sensitivity and specificity analysis (**cyclic topology**)*. **Panel 1:** Curve plot shows mean sensitivity as a function of the mean false-positive rate (*1-specificity*) across all ground-truth edges. Curves marked with circles indicate the different TFNBS *E/H* parameter value combinations tested. Each curve represents a different E parameter, and each point along the curves indicates a different value of *H*. Some parameters (*E* = 1 combined with *H* < 3.5) yielded specificities < 0.997 and are not shown. Curves marked with triangles or diamonds indicate values obtained with the two tested variants of the NBS, at the F-thresholds that displayed specificities > 0.997 (9.5 through 16). **Panel 2:** Curves show mean sensitivities for each of the five ground-truth connected components as a function of the mean overall false-positive rate, for the four *E* parameter values assessed across different H values. Components' contrast-to-noise ratios (CNR) are indicated. The y axes have been rescaled according to the range of values displayed.

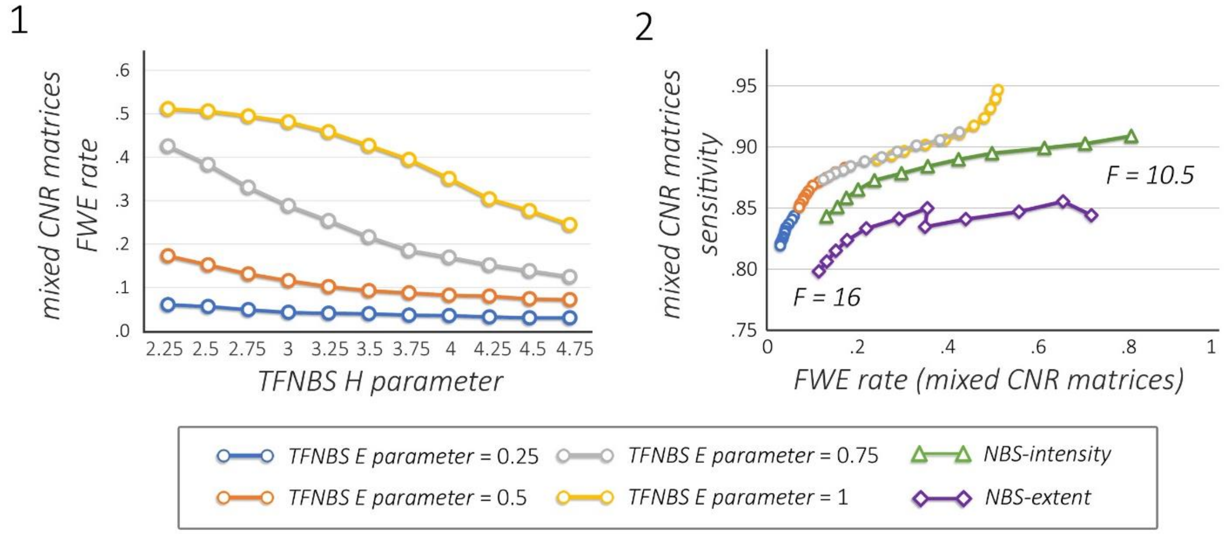

*Supporting Information, Figure 4. TFNBS and NBS family-wise error rates (***cyclic** ***topology****).* **Panel 1** shows mean family-wise error (FWE) rates obtained using TFNBS with *mixed-CNR* cyclic *topology matrices*. Different curves depict different *E* parameter values, as a function of *H* parameter values. **Panel 2:** relationship between sensitivity and FWE rates, obtained with mixed-CNR matrices and *tree* topology. Curves marked with circles represent different TFNBS *E/H* parameter combinations, and curves marked with triangles or diamonds indicate values obtained with NBS using 12 F thresholds (10.5 through 16) that yielded similar FWE rates.

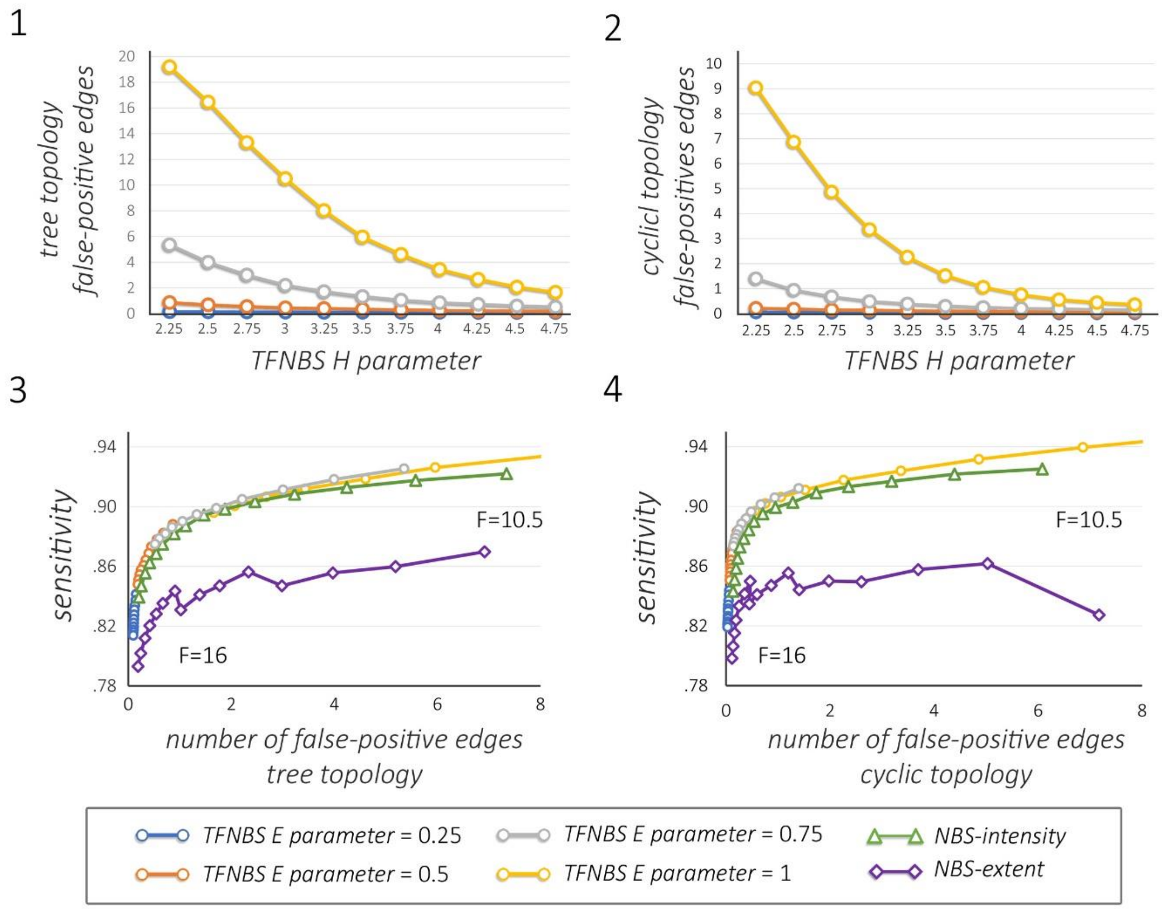

Supporting Information, Figure 5. Number of false-positive findings. Figures display the number of false-positive edges identified across the connectome for every comparison in which at least one false positive was observed. **Panel 1** shows the results obtained with TFNBS and mixed-CNR *tree*-topology matrices. Different curves represent results for different *E* parameter values, as a function of *H* parameter values. **Panel 2** shows the results obtained with TFNBS and mixed-CNR *cyclic*-topology matrices. **Panels 3 and 4** depict the relationship between sensitivity and number of false-positive edges for the *tree* topology (Panel 3) and *cyclic* topology (Panel 4). Curves marked with circles indicate the different TFNBS *E/H* parameter value combinations tested. Each curve represents a different *E* parameter, and each point along the curves indicates a different value of *H*. Curves marked with triangles or diamonds indicate values obtained with the two tested variants of the NBS, at F-thresholds between 9.5 and 16.

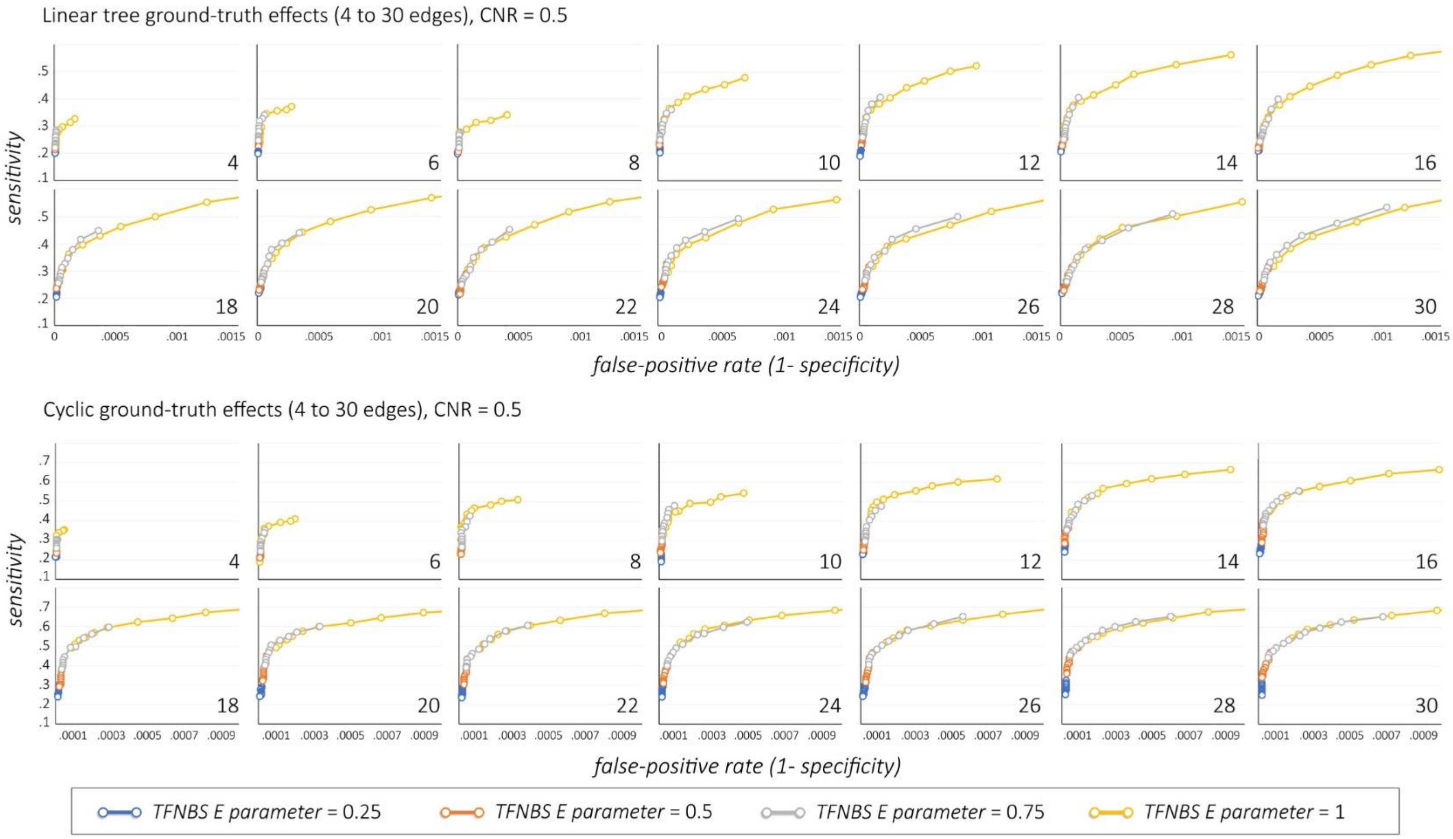

Supporting Information, Figure 6. Growing ground-truth components, contrast-to-noise ratio (CNR) = 0.5 – sensitivity and specificity analysis. Random-noise matrices containing ground-truth connected components of sizes ranging from four to 30 edges (at steps of two) were assessed, with two topologies (linear *tree* topology, top, and *cyclic* topology, bottom).

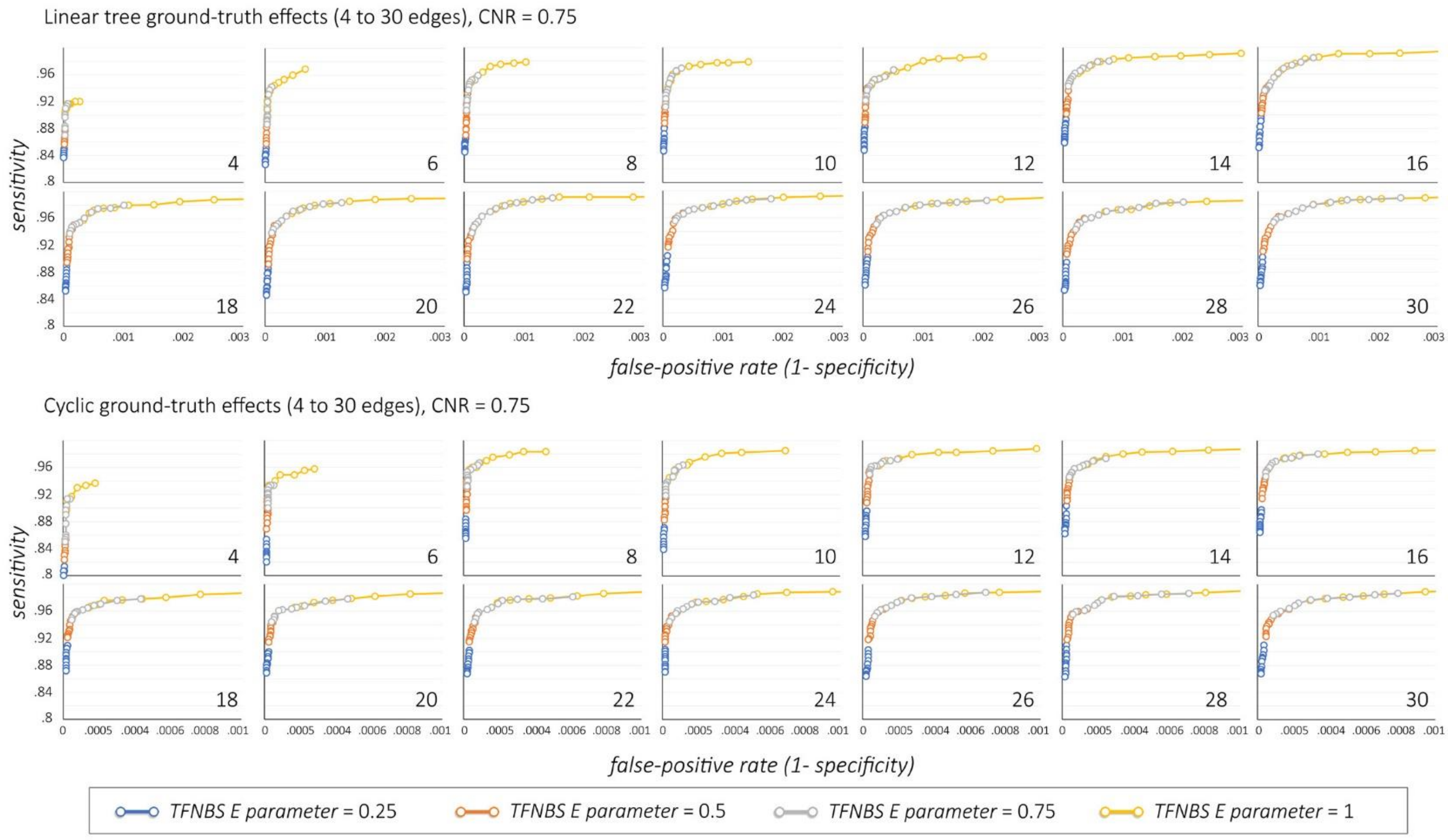

Supporting Information, Figure 7. Growing ground-truth components, contrast-to-noise ratio (CNR) = 0.75 – sensitivity and specificity analysis. Random-noise matrices containing ground-truth connected components of sizes ranging from four to 30 edges (at steps of two) were assessed, with two topologies (linear *tree* topology, top, and *cyclic* topology, bottom).
